# Home care needs assessment among caregivers of children and adolescents with osteogenesis imperfecta: a cross-sectional study

**DOI:** 10.1186/s12875-024-02367-8

**Published:** 2024-04-19

**Authors:** Xinyi Wang, Yuqing Li, Yaping Zhong, Min Wang, Xuehua Liu, Wenxuan Han, Huifang Chen, Ji Ji

**Affiliations:** 1 School of Nursing, Shandong First Medical University & Shandong Academy of Medical Sciences, No.6699 Qingdao Road, Huaiyin District, Jinan, Shandong Province 250117 China; 2 Department of Pediatric Orthopaedics, Shandong Provincial Hospital Affiliated to Shandong First Medical University& Shandong Academy of Medical Sciences, No.324 Five Weft Seven Road, Huaiyin District, Jinan, Shandong Province 250021 China; 3 School of Nursing, Shandong First Medical University & Shandong Academy of Medical Sciences, No.619 Changcheng Road, Daiyue District, Taian, Shandong Province 271016 China; 4 Academic Nursing Unit, Peter MacCallum Cancer Centre, No.305 Grattan Street, Melbourne, VIC 3000 Australia; 5 School of Nursing, Guangzhou Medical University, No.195 Dongfengxi Road, Guangzhou, Guangdong 510182 China; 6 Department of Nursing, Shandong Provincial Hospital Affiliated to Shandong First Medical University& Shandong Academy of Medical Sciences, No.324 Five Weft Seven Road, Huaiyin District, Jinan, Shandong Province 250021 China

**Keywords:** Adolescents, Children, China, Home Care needs, Osteogenesis Imperfecta

## Abstract

**Background:**

Children and adolescents with complex medical issues need home care services; however, few studies have provided insight into the unmet home care needs of the families of patients with osteogenesis imperfecta (OI). In this study, we aimed to assess the home care needs of caregivers of children and adolescents with OI and the associated factors.

**Methods:**

A self-administered questionnaire was administered online to 142 caregivers of patients with OI aged 3–17 years between May and October 2022 from 25 provinces in China. The questionnaire comprised 15 questions on demographic variables and 14 questions on home care needs. Chi-square analysis was used to compare group differences for categorical variables. Multivariate binary logistic regression analysis was conducted to examine predictors of caregivers’ home care needs.

**Results:**

The study findings indicated that 81.5% of caregivers had high home care needs. The three leading types of home care needs were helping the child carry out physical fitness recovery exercises at home (72.5%), understanding precautions regarding treatment drugs (72.5%), and relieving the child’s pain (70.4%). OI patients’ poor self-care ability (adjusted odds ratio = 5.9, 95% confidence interval = 1.8–19.0*)* was related to caregivers’ high level of home care needs.

**Conclusions:**

The findings of this study suggest that future scientific research and nursing guidance should focus on OI patients’ physical training, medication management, pain relief, fracture prevention, and treatment. In addition, caregivers of patients with poor self-care ability should receive special attention in the development of interventions. This study can help with addressing the unmet home care needs of caregivers of children and adolescents with OI. It is vital to develop a personalized intervention plan based on patients’ self-care ability.

**Supplementary Information:**

The online version contains supplementary material available at 10.1186/s12875-024-02367-8.

## Background

Osteogenesis imperfection (OI) is a rare genetic disease occurring in 1 in 15–20,000 births [1]. OI is characterized by bone fragility and skeletal deformity [2]. Currently, the treatment for OI mainly includes preventing fractures, controlling symptoms, and increasing bone mass and is divided into non-surgical and surgical procedures [1]. Although various treatments have a positive therapeutic effect, the disease remains incurable. The primary goal of therapy is to maximize patients’ physical function, mobility, and social participation [3]. The earliest diagnosis of OI is during the first to early second trimester [4]. Throughout the disease course, the fracture risk among patients with OI is highest in childhood and adolescence [5]. A prior study indicates that managing and treating OI during this period is crucial for the development of motor skills and independence later in life [3].

Parents and caregivers of pediatric patients with chronic conditions, such as OI, have an important role in ensuring safety, effectiveness, and supportive care [6]. Caregivers of patients with OI require support [7], especially during patients’ adolescent period [8], including fracture management support [7, 9–13], home rehabilitation support [7], pain management support [7, 10, 12], daily care support [10], psychological support [11], and disease information support [14]. Childhood and adolescence can be challenging for the families of patients with OI [8], and caregivers experience considerable stress [15]. Caregivers must help patients not only with physical conditions such as fractures, pain, surgery, and mobility [7, 10, 13] but also with loneliness and feelings of inferiority caused by forced interruptions in schooling and discrimination [8]. OI requires an interdisciplinary care strategy, facilitating a continuous care process through collaboration among physicians, physical therapists, nurses, geneticists, and other professionals [16]. Nurses hold a unique position in fostering and nurturing partnerships between care providers, patients, and families [17]. Nurses can serve as communication hubs between caregivers and the care team [17], taking responsibility for understanding caregivers’ daily care needs and educating them to perform hands-on care tasks [18].

As early as 2002, a Swedish study reported that it was difficult for OI families to obtain disease information and support [19]. Subsequently, Dogba et al. [9] found that the care needs in caring for adolescents with OI involved diagnosis, safety, fractures, surgery, pain, and activities. Several approaches have been proposed to address these issues. A prior study confirmed that bisphosphonate therapy can improve the living conditions of OI families [20]. In addition, OI patients can gain sufficient information through 10 semi-structured, 3-hour psycho-educational training sessions, as demonstrated in a 2014 study in Turkey [21].

Over the past 5 years, several studies have explored the care needs of the families of patients with OI, yet many challenges remain unresolved [7, 8]. Insufficient guidance for OI patients may be contributing to this issue. Recent qualitative studies conducted in Canada have found that patients with OI have had difficulty accessing reliable disease information before 2020 [10, 22]. Furthermore, children with OI struggle have limited access to pediatric occupational therapy, physiotherapy, and appropriate equipment. In some cases, health professionals with limited experience in treating OI may provide incorrect disease knowledge and treatment recommendations; this was confirmed in a 2022 study conducted in the United Kingdom [8]. In addition, a systematic review demonstrated that medical professionals often lack sufficient knowledge about rare diseases like OI unless they specialize in a particular area [13].

Given the above issues, improving the efficiency and accessibility of care deserves special attention. According to a study conducted in China including 802 adults and children with OI, patients lack sufficient knowledge about health self-management [23]. The study also identified female patients, those over 40 years or under 18 years of age, and patients in junior middle school as having a higher risk of poor self-care [23]. However, few studies have examined the impact of socioeconomic factors on care needs. A 2019 systematic review showed that fear of fractures is a critical issue affecting OI patients and their families [13]. However, the degree of attention given by caregivers to other care needs remains unknown. A needs assessment can determine priorities for the most effective use of medical resources [24] and can be used to establish priorities for nursing instruction, which may improve the life status of OI families in a shorter period. Currently, it is estimated that there are 5 million people with OI worldwide, including approximately 100,000 in China. Thus, evaluating and resolving the home care needs of OI patients in China may serve as a reference for improving the family life status of the OI population globally.

In this study, we aimed to use questionnaires to assess the specific areas in which caregivers of children and adolescents with OI need help to highlight directions for nursing guidance. The primary focus is on assessing and ranking the importance of various home care needs and identifying associated factors. The findings of this study are expected to provide evidence for identifying gaps in the home care needs of OI families and establishing priorities for nursing instruction.

## Methods

### Design and participants

This was a cross-sectional survey among caregivers of children and adolescents with OI in China. In 2011, the National Bureau of Statistics divided China into four regions based on economic development levels: Northeast, East, Central, and West. This study used a stratified random sampling method to select 25 provinces from these four regions^1^. All participants were recruited by snowball sampling. Initially, we randomly selected one caregiver of a child or adolescent with OI from each of the 25 provinces. These caregivers were registered in the China-Dolls Center for Rare Disorders of the Illness Challenge Foundation^2^. Before formal data collection, we discussed unclear items on the questionnaire with the 25 participants to ensure clarity and understanding of the questionnaire language. Next, the 25 caregivers shared the questionnaire with two caregivers of a child or adolescent with OI whom they knew, and the second group shared the questionnaire in the same way.

According to Kendall’s sample size calculation principle [25], the ideal sample size should be 5–10 times the number of variables. This study had 16 variables (15 demographic variables and 1 home care need item). Considering an attrition rate of 20%, the target sample size was 96–192. Of the 300 questionnaires distributed, 118 were returned. After excluding invalid questionnaires, a total of 142 completed surveys comprised the dataset (response rate: 47%).

The inclusion criteria for survey respondents were (1) caregivers of a child or adolescent aged 3–17 years who was diagnosed with OI by a pediatric orthopedic surgeon; (2) the respondent was the patient’s primary caregiver; (3) care time ≥ 6 months, caregiver age ≥ 18 years, and caregiver knew the patient’s disease status. The exclusion criteria were (1) patients with other serious diseases that might affect their home care needs, such as severe heart, kidney, and hepatobiliary diseases; (2) patients with mental disorders; (3) caregivers with cognitive difficulties or psychiatric disorders. This study received approval from the Ethical Committee of Shandong Provincial Hospital Affiliated with Shandong First Medical University (number SWYX: NO.2022 − 518). An informed consent letter was signed by caregivers who agreed to participate in the study.

### Data collection

The questionnaire was uploaded to the professional online questionnaire platform Questionnaire Star (http://www.wenjuan.com/), generating a link to the questionnaire. All participants were recruited between May and October 2022. Potential participants were provided with a link or QR code via the social media platform WeChat. Each Internet protocol (IP) address could only be used once to complete the questionnaire.

### Study instrument

**Fig. 1:**
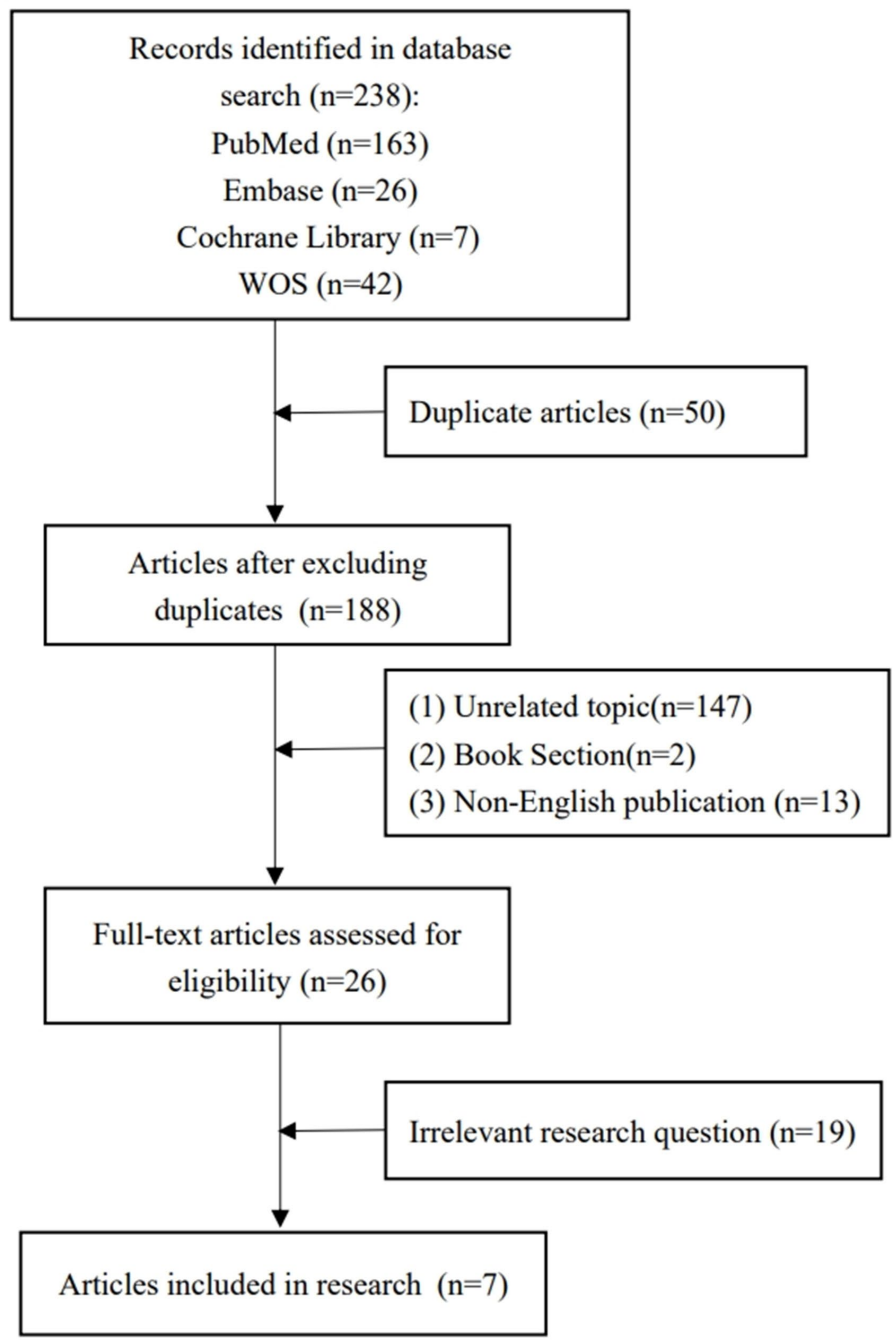
Study selection process

The Caregivers of Osteogenesis Imperfecta Children and Adolescents Home Care Needs Questionnaire (Questionnaire.pdf), developed by the research team, was used in this study. In the first step, we extensively reviewed research on the impact on families of OI. The search was from database inception up to December 2021. The following search terms were used: “Osteogenesis Imperfecta / Brittle Bone Disease / Fragility Ossium / Lobstein Disease,” and “Home Nursing / Home Care / Family Nursing / Family Care / Family-Centered.” The following electronic databases were searched: PubMed, Embase, Cochrane Library, and WOS. We finally included seven studies [7, 9–14]. See Fig. 1 for the study selection process.

The literature review revealed that caregivers’ main support needs in caring for OI patients were fracture [7, 9–13], surgery [10], pain [7, 10, 12], safety [9, 12, 13], exercise [7, 10], assistive devices [12, 14], psychological [11, 13], and routine care (medication, dressing, diet, and cleaning) [10]. Based on this, we summarized care needs into 14 items to reflect the level of caregiver’s home nursing needs more concisely and clearly.

Fracture corresponds to the items “Improving the child’s attitude about fracture prevention” and “Self-rescue knowledge after fractures.” Surgery corresponds to the items “Helping the child cope with postoperative complications of fractures” and “Caring for the child after plaster external fixation.” Pain corresponds to the item “Relieving the child’s pain”; safety corresponds to “Adapting my house so as to be safer”; exercise corresponds to “Helping the child carry out physical fitness recovery exercises at home.” Assistive device corresponds to the item “Use of assistive devices (cane, wheelchair, walker)”; psychological corresponds to “Relieving psychological and mental stress.” Routine care corresponds to the items “Understanding precautions regarding treatment drugs,” “Helping the child have proper body posture,” “Preparing a healthy daily diet for the child,” “Helping the child manage personal hygiene,” and “Helping the child put on and take off clothes.”

Following that, we invited six experts in relevant fields to assess the content validity, calculating the content validity index. The six experts comprised four pediatric orthopedic clinical physicians and nurses, one medical school professor, and one statistics expert. Among them, two experts hold bachelor’s degrees, two hold master’s degrees, and two hold doctoral degrees. The questionnaires were delivered to the experts via Questionnaire Star, and the data of the six experts were entered into Microsoft Excel 2019 for analysis. The item content validity index (I-CVI) ranged from 0.83 to 1, and the scale content validity index (S-CVI/Ave) was 0.93. Finally, to evaluate the reliability of home care needs items, Cronbach’s alpha was calculated using IBM SPSS 26.0. The questionnaire showed good internal consistency, with a Cronbach’s alpha score based on all caregivers who participated (*N* = 142) = 0.948. Taken together, this showed that the questionnaire had good validity and reliability.

The final version of the survey comprised 15 questions on demographic variables and 14 questions on caregivers’ home care needs. Participants indicated their level of home care needs for each item on a 5-point Likert scale: 1 = No help needed at all, 2 = Help rarely needed, 3 = Help sometimes needed, 4 = Help very much needed, 5 = Help extremely needed. The scale score was calculated as the average score of each item, and the range was 1–5. A higher score indicated higher home care needs.

### Data analysis

All data from the questionnaire were automatically collected using Questionnaire Star and imported into Excel 2019. IBM SPSS version 26 was used for statistical analyses. Invalid questionnaires were excluded according to the following criteria: (1) incomplete demographic information for ≥ 5 items; and (2) demographic data did not meet the inclusion criteria. Demographic characteristics and home care need variables were descriptively analyzed; categorical variables are presented as frequencies and percentages.

An average score ≤ 3 (“No help needed at all,” “Help rarely needed,” and “Help sometimes needed”) was defined as lower home care needs and assigned a value of 0. An average score > 3 (“Help very much needed” and “Help extremely needed”) was defined as higher home care needs and assigned a value of 1. Participants’ scores for each item and average scores on the overall questionnaire were categorized according to the above standards, with scores > 3 indicating high needs and scores ≤ 3 indicating lower needs. Chi-square analysis was used to compare group differences between categorical variables in home care needs. Multivariable binary logistic regression was conducted to estimate the related factors of caregivers’ home care needs, and the Enter method was applied. We selected the reference categories based on the mean home care needs score for each variable category; the reference group was the category with the lowest score. According to sociodemographic differences, variables with *P* ≤ 0.05 were included in multivariate regression analysis. The patient’s age was considered a potential confounder and was also included in the multivariate regression analysis to minimize its impact. A 95% confidence interval (CI) was used to determine the level of significance.

## Results

### Demographic characteristics

Respondents’ demographic information is shown in Table 1. This study included 142 caregivers of children and adolescents with OI. The mean age of patients was 9.2 years (± 4.3 years). Most caregivers were the patient’s mother (61.3%). Most patients had OI for over 4 years (65.5%), and 72 (50.7%) patients were categorized as having complete/basic self-care abilities. More than half (56.3%) of respondents were from rural areas, and 47 (33.1%) families of OI patients were in the low-income group (≤ 2000 RMB). Most participants (83.8%) had health insurance but did not have subsistence allowances (71.8%) or disability subsidies (64.1%).

**Table 1:** Demographic characteristics (*N* = 142)

Variable	n	%
**Patients**		
Sex		
Male	77	54.2
Female	65	45.8
Age, y		
3–6	53	37.3
7–12	51	35.9
13–17	38	26.8
Place of residence		
Urban/suburban	62	43.7
Rural	80	56.3
Self-care ability*		
Limited self-care ^a^	70	49.3
Complete/basic self-care ^b^	72	50.7
Fractures in the past year		
≤ 1	74	52.1
> 1	68	47.9
Family history		
Yes	44	31.0
No	98	69.0
Illness duration, y		
≤ 2	15	10.6
2–4	34	23.9
≥ 4	93	65.5
Schooling status		
At school	77	54.2
At home	65	45.8
Health insurance		
Medical insurance for urban employees	9	6.3
Medical insurance for urban and rural residents	110	77.5
Self-pay	23	16.2
Subsistence allowances		
Yes	40	28.2
No	102	71.8
Disability subsidies		
Yes	51	35.9
No	91	64.1
**Caregiver**		
Identity		
Father	46	32.4
Mother	87	61.3
Sibling/missing data	9	6.3
Marital status		
Unmarried/divorced/widowed	23	16.2
Married	119	83.8
Education level		
Below middle school	33	23.2
Middle school	71	50.0
Above middle school	38	26.8
Monthly household income, RMB		
low income (≤ 2000)	47	33.1
middle income (2001–5000)	57	40.1
higher income (5001–10,000)	18	12.7
high income (> 10,000)	20	14.1

*Self-care is defined as essential tasks of taking care of oneself such as eating, dressing, grooming, and management of oral and toilet hygiene. ^a^ Limited self-care: completely unable to care for oneself/need uninterrupted assistance from another person; ^b^ Complete/basic self-care: completely able to care for oneself/able to care for oneself but it is laborious and time-consuming/need occasional assistance from another person

### Home care needs of caregivers of children and adolescents with OI

To understand the areas where caregivers of children and adolescents with OI need help to provide more targeted guidance, we ranked the scores for each item. Among 142 caregivers, 81.5% of caregivers had an average score > 3, indicating relatively higher home care needs, in comparison with caregivers with an average score of ≤ 3 for home care needs. The three leading home care needs items were “Helping the child carry out physical fitness recovery exercise at home” (72.5%), “Understanding precautions regarding treatment drugs” (72.5%), and “Relieving the child’s pain” (70.4%). More detailed information is shown in Table 2.

**Table 2:** Home care needs of caregivers of children and adolescents with OI (*N* = 142)

Items	n (%) of higher needs	Order
**Home care needs**	115/142 (81.5%)	
Helping the child carry out physical fitness recovery exercises at home	103/142 (72.5%)	1
Understanding precautions regarding treatment drugs	103/142 (72.5%)	1
Relieving the child’s pain	100/142 (70.4%)	3
Improving the child’s attitude toward fracture prevention	98/142 (69.0%)	4
Helping the child have proper body posture (standing, sitting)	97/142 (68.3%)	5
Self-rescue after a fracture	96/142 (67.6%)	6
Helping the child cope with postoperative complications of fractures	96/142 (67.6%)	6
Relieving psychological and mental stress	89/142 (62.7%)	8
Caring for the child after plaster external fixation	87/142 (61.3%)	9
Use of assistive devices (cane, wheelchair, walker)	76/142 (53.5%)	10
Adapting my house to be safer	70/142 (49.3%)	11
Preparing a healthy daily diet for the child	69/142 (48.6%)	12
Helping the child manage personal hygiene	44/142 (31.0%)	13
Helping the child put on and take off clothes	43/142 (30.3%)	14

### Comparison of demographic characteristics among home care needs

Chi-square analysis was used to compare group differences between categorical variables. Table 3 shows all variables with *P* ≤ 0.05. Patients’ self-care ability (*P* < 0.001) and number of fractures in the past year (*P* = 0.003) were significantly different in terms of caregivers’ home care needs. The following variables were entered into the multivariate binary logistic regression models, which were used to control for potential confounding factors. In addition to the above variables, patients’ age was included in the statistical analysis to minimize its impact.

**Table 3:** Comparison of demographic characteristics among home care needs (*N* = 142)

Variables	n	χ^2^	P
Patient self-care ability			
Limited self-care	70	15.858	< 0.001*
Complete/basic self-care	72		
Fractures in the past year			
≤ 1	74	8.800	0.003*
> 1	68		

* Chi-square analysis: statistical significance at *P* ≤ 0.05

### Multivariable binary logistic regression models

There were no violations of logistic regression analysis assumptions in the multivariate model, with no multicollinearity (VIF < 10.0) or outliers identified (studentized residuals < 2.5). The percentage correct was 81.0% and the receiver operating characteristic value was 0.771 (95% CI = 0.679–0.863). The multivariate model reported was significant (*P* < 0.001), with acceptable goodness-of-fit statistics (Hosmer–Lemeshow: *P* = 0.850), explaining a variance of 22.4% (Nagelkerke R2). The results in Table 4 show that patients’ self-care ability was the factor most significantly associated with caregivers’ home care needs (AOR = 5.9, 95%CI = 1.8–19.0).

**Table 4:** Multiple binary logistic regression analysis of factors related to home care needs (*N* = 142)

Characteristic	Predictor	B	SE	Wald	df	Exp(B)	95% CI	P Value
Patient age, y	13–17					Reference		
	3–6	0.16	0.56	0.08	1	1.2	0.4–3.5	0.782
	7–12	0.47	0.57	0.68	1	1.6	0.2–4.9	0.410
Patient self-care ability	Complete/basic self-care					Reference		
	Limited self-care	1.78	0.59	8.95	1	5.9	1.8–19.0	0.003*
Fractures in the past year	≤ 1					Reference		
	> 1	0.92	0.53	2.97	1	2.5	0.9–7.1	0.085

* Indicates statistical significance at *P* ≤ 0.05

## Discussion

To provide more efficient and targeted guidance for the families of patients with OI, we aimed to assess the home care needs of caregivers of children and adolescents with OI and the associated factors. In our study, 81.5% of caregivers had an average score > 3 for home care needs, indicating an extreme need for home care support. Areas where caregivers had the greatest need for help were patients’ exercise, treatment drugs, and pain management. OI patients’ poor self-care ability was related to higher home care needs among caregivers, according to multivariate analysis.

Most respondents had higher home care needs, indicating the deficiency of self-care education for caregivers of pediatric patients with OI in China. Among caregivers, 72.5% needed nursing instruction on how to help the child carry out physical fitness recovery exercises at home and understanding precautions regarding treatment drugs, which were the highest among all care need items. This result is unsurprising in that treatment drugs are widely used to improve the symptoms of OI in patients [2]. As early as the 1990s, studies have investigated the use of bisphosphonates in children with severe OI [26], Today, bisphosphonates are considered the standard therapy for children with OI [1]. A systematic review revealed that intravenous bisphosphonate therapy can improve OI patients’ mobility [27]. However, the main disadvantage of bisphosphonates is that their effectiveness is relatively weak, and acute side effects such as fever, diarrhea, dysautonomia storm, hypocalcemia, and hypophosphatemia are also observed [1]. Precautions regarding treatment drugs require that explicit guidance and assistance be given to caregivers of children and adolescents with OI.

As mentioned above, most caregivers needed nursing instructions on helping the patient carry out physical fitness recovery exercises at home. As emphasized in a previous study, it is mandatory to maintain OI patient’s physical activity through a tailored physical training program, and the training should preferably be done in the patient’s environment [28]. A recent study revealed that the life quality and musculoskeletal functionality of inactive OI patients improve when they start some type of physical exercise [16]. A consensus statement on physical rehabilitation in children and adolescents with OI, developed at the 13th International Conference on OI in August 2017, emphasized that early training in muscle strength, joint mobility, and limb function; using assistive devices appropriately; and avoidance of overprotection is needed [3]. The statement can guide the development of physical training programs to a certain extent, but relevant studies are still lacking, with only four studies being randomized or longitudinal studies [29–32]. Therefore, future additional longitudinal studies are needed to develop personalized physical training programs for patients with OI in a multidisciplinary manner.

The high need among caregivers to relieve the patient’s pain lends support to previous evidence that nearly all pediatric patients with OI experience pain, which interferes with their normal life [33]. Pain is a well-recognized symptom in children and adolescents with OI [34]. Caregivers of children with OI who have higher levels of pain also have a higher risk of increased stress [15]. A literature review identified that multimodal treatments can be effective at relieving pain in children with OI, including bisphosphonate therapy, surgical intervention, physical therapy, and psychosocial support [35]. However, there are no guidelines summarizing how to conduct pain assessment and management in this population. Thus, further research is warranted to guide caregivers in helping children and adolescents with OI through proper pain management.

It is unsurprising that caregivers of children and adolescents with OI tend to be concerned with how to improve the patient’s attitude regarding fracture prevention, more so than self-rescue knowledge after a fracture. This finding is also consistent with previous results that OI patients and caregivers pay extra attention to how to avoid fractures [36]. Caregivers have a fear of fractures [3] and attempt to avoid fractures by protecting the patient [13]. Caregivers are also affected by advice from doctors to avoid excessive exercise for safety reasons [23]. A combination of the above factors may explain the tendency toward overprotection among caregivers and patients. However, it must also be noted that the risk of fracture cannot be completely eliminated and overprotection is not recommended as this can be disadvantageous to rehabilitation [7, 36]. At present, there are no definitive guidelines for fracture prevention and management in OI patients. It is therefore necessary to develop comprehensive and practical clinical guidelines for these patients.

Poor self-care ability among patients with OI was the predominant predictor of caregivers’ higher home care needs, according to multivariate analysis. This is in line with the findings of Lazow et al. [15] that parents who care for children with OI who have lower physical functioning are under greater pressure, which may partly explain their higher home care needs. Furthermore, this result may indicate that caregivers’ level of emphasis on poor self-care ability resulting from fractures in OI patients may surpass the seriousness of the fracture itself. Multiple studies [8, 22] have shown that patients with OI are eager to achieve an independent and normal life. Therefore, greater attention should be given to caregivers of OI patients with poor self-care ability when tailoring health educational interventions. It is urgent to improve the self-care ability of the population with OI through high-quality home care instruction.

Socioeconomic factors such as sex, age, place of residence, monthly income, social support, and caregivers’ educational level did not significantly affect caregivers’ home care needs in this study. Consistent with earlier research findings in the context of China, sex and place of residence [37] had no significant effect on quality of life in patients with OI. Moreover, a cross-sectional study by Vanz et al. in southern Brazil [38] found that quality of life among caregivers of children and adolescents with OI was not associated with economic status. Conversely, another study by Vanz et al. [39] showed that quality of life was correlated with the age of OI patients. The impact of OI on the family’s quality of life and income has also been demonstrated [37]. According to the above related research, the influence of socioeconomic factors on OI families remains unclear, with only a few related studies. It is important to confirm the results of the current study in a prospective, randomized, controlled trial in the future to more accurately identify the population characteristics of OI families with high home care needs.

Although socioeconomic factors did not significantly impact the home care needs of caregivers of children and adolescents with OI in this study, it is noteworthy that most participants’ monthly family income was less than 5000 RMB and most families did not have subsistence allowances or disability subsidies, according to the descriptive analysis. This might suggest that China is paying insufficient attention to the life quality and healthcare support of patients with rare diseases, such as OI. Further refinement to improve the existing social security system is necessary to satisfy OI families’ basic living and medical requirements.

### Limitations

This study has several limitations. First, although we recruited participants from 25 provinces in China in this survey, the larger proportion of participants with lower socioeconomic status may have an impact on the generalizability of the results. In addition, the questionnaire used in this study has good reliability and validity; however, it is necessary to expand the sample size in the future and conduct convergence/diversity validity analyses to enhance the generalizability of the questionnaire and results. Second, the online questionnaire was distributed using snowball sampling through the WeChat platform. Those who completed the questionnaire were likely to be interested in home care for patients with OI, which may result in a certain degree of selection bias. Finally, patients’ self-care ability was self-reported rather than professionally assessed. Although we provided explanations of the various levels of self-care to caregivers in the questionnaire, a certain risk of bias remains. A more accurate assessment of OI patients’ self-care ability is needed in future studies to confirm the present results.

## Conclusion

In our study, 81.5% of caregivers of children and adolescents with OI showed higher home care needs, with patients’ self-care ability being the predominant predictor of high care needs. The following practical conclusions were obtained in this study. First, future scientific research and nursing guidance should focus on OI patients’ physical training, medication management, pain relief, and fracture prevention and treatment to improve the efficiency and accessibility of care. Second, caregivers of patients with poor self-care ability should receive particular attention in the development of interventions. Finally, further efforts should be devoted to improving the current social security system to satisfy the basic life and medical needs of the families of patients with OI.

### Electronic supplementary material

Below is the link to the electronic supplementary material.

Supplementary Material 1

## Data Availability

All of the data generated during and/or analyzed during the current study are available from the corresponding author on reasonable request.

## Footnotes

1 Northeast: Jilin, Liaoning; East: Beijing, Tianjin, Hebei, Shanghai, Jiangsu, Zhejiang, Shandong, Fujian; Central: Shanxi, Anhui, Jiangxi, Henan, Hubei; West: Guangxi, Chongqing, Sichuan, Guizhou, Yunnan, Shanxi, Gansu, Qinghai, Ningxia, Xinjiang.

2 The Illness Challenge Foundation is the first public welfare foundation focused on rare diseases in Beijing. China-Dolls Center for Rare Disorders was established in 2008 with a mission to provide medical, lifestyle, educational, and employment support for patients with OI. The center has registered over 4,000 families of OI patients.

## Abbreviations

OI: Osteogenesis imperfecta
