# Supplementary material for: Home care needs assessment among caregivers of children and adolescents with osteogenesis imperfecta: a cross-sectional study
Source: BMC Prim Care. 2024 Apr 19;25:119. doi: 10.1186/s12875-024-02367-8 (PMC11027538; doi:10.1186/s12875-024-02367-8)
Supplement: Supplementary file 1 — Supplementary Material 1 [file 12875_2024_2367_MOESM1_ESM.docx]

**Caregivers of Osteogenesis Imperfecta Children and Adolescents Home Care Needs Questionnaire**

**General Data Survey Form**

**Patients**

11. Disability subsidies

a) With

b) Without

**Caregivers**

1. Identity

a) Father

b) Mother

c) Sibling

2. Marital status

a) Unmarried/divorced/widowed

b) Married

3. Education level

a) Below middle school

b) Middle school

c) Above middle school

4. Monthly household income, RMB

a) low income (≤2000)

b) middle income (2001–5000)

c) higher income (5001–10000)

d) high income (>10000)

*Self-care is defined as essential tasks of taking care of oneself such as eating, dressing, grooming, and management of oral and toilet hygiene.

a Limited self-care: completely unable to care for oneself/need uninterrupted assistance from another person;

b Complete/basic self-care: completely able to care for oneself/able to care for oneself but it is laborious and time-consuming/need occasional assistance from another person.

1. Sex

a) Male

b) Female

2. Age, y

a) 3-6

b) 7-12

c) 13-17

3. Place of residence

a) Urban/suburban

b) Rural areas

4. Self-care ability*

a) Limited self-care ^a^

b) Complete/basic self-care ^b^

5. Fracture times in the past year

a) ≤1

b) >1

6. Family history

a) Yes

b) No

7. Illness duration, y

a) ≤ 2

b) 2-4

c) ≥ 4

8. Schooling status

a) At school

b) At home

9. Health insurance

a) Medical insurance for urban employees

b) Medical insurance for urban and rural residents

c) Self-pay

10. Subsistence allowances

a) With

b) Without

**Caregivers needs Survey Form**

Please circle your answers according to how much help is currently needed. 1 Help not at all needed

2 Help rarely needed

3 Help sometimes needed

4 Help very much needed

5 Help extremely needed

| How to help my child carry out physical fitness recovery exercises at home | 1 2 3 4 5 |
| --- | --- |
| Helping the child carry out physical fitness recovery exercises at home | 1 2 3 4 5 |
| Understanding precautions regarding treatment drugs | 1 2 3 4 5 |
| Relieving the child’s pain | 1 2 3 4 5 |
| Improving the child’s attitude toward fracture prevention | 1 2 3 4 5 |
| Helping the child have proper body posture (standing, sitting) | 1 2 3 4 5 |
| Self-rescue after a fracture | 1 2 3 4 5 |
| Helping the child cope with postoperative complications of fractures | 1 2 3 4 5 |
| Relieving psychological and mental stress | 1 2 3 4 5 |
| Caring for the child after plaster external fixation | 1 2 3 4 5 |
| Use of assistive devices (cane, wheelchair, walker) | 1 2 3 4 5 |
| Adapting my house to be safer | 1 2 3 4 5 |
| Preparing a healthy daily diet for the child | 1 2 3 4 5 |
| Helping the child manage personal hygiene | 1 2 3 4 5 |
